# The Effect of High-Fat Diet-Induced Obesity on the Expression of Nutrient Chemosensors in the Mouse Stomach and the Gastric Ghrelin Cell

**DOI:** 10.3390/nu12092493

**Published:** 2020-08-19

**Authors:** Maria Nunez-Salces, Hui Li, Stewart Christie, Amanda J. Page

**Affiliations:** 1 Vagal Afferent Research Group, Adelaide Medical School, The University of Adelaide, Adelaide, SA 5005, Australia; maria.nunez@adelaide.edu.au (M.N.-S.); hui.li01@adelaide.edu.au (H.L.); stewart.christie@adelaide.edu.au (S.C.); 2 Centre of Research Excellence in Translating Nutritional Science to Good Health, Adelaide Medical School, The University of Adelaide, Adelaide, SA 5005, Australia; 3 Nutrition, Diabetes & Gut Health, Lifelong Health Theme, South Australian Health & Medical Research Institute (SAHMRI), Adelaide, SA 5000, Australia

**Keywords:** ghrelin, stomach, nutrient sensing, CaSR, obesity, high-fat diet

## Abstract

The stomach is the primary source of the orexigenic and adiposity-promoting hormone, ghrelin. There is emerging evidence on the nutrient-mediated modulation of gastric ghrelin secretion. However, limited information is available on gastric nutrient-sensing mechanisms in high-fat diet (HFD)-induced obesity. This study investigated the impact of HFD-induced obesity on the expression of nutrient chemosensors in mouse stomach, particularly ghrelin cells. Male C57BL/6 mice were fed either a standard laboratory diet (SLD) or HFD for 12 weeks. The expression of ghrelin, enzymes involved in ghrelin production (PC1/3, GOAT) and nutrient chemosensors (CD36, FFAR2&4, GPR93, CaSR, mGluR4 and T1R3) was determined by quantitative RT-PCR in the mouse corpus and antrum. Immunohistochemistry assessed the protein expression of CaSR and ghrelin in the corpus and antrum. Antral mRNA levels of CaSR and PC1/3 were increased in HFD compared to SLD mice, while mRNA levels of all other nutrient chemosensors examined remained unchanged. CaSR immunolabelling was observed in the gastric antrum only. Nearly 80% of antral ghrelin cells expressed CaSR, with a similar cell density and co-expression in SLD and HFD mice. In conclusion, HFD-induced obesity increased CaSR mRNA expression in mouse antrum. However, the high antral co-expression of CaSR and ghrelin was unaltered in HFD compared to SLD mice.

## 1. Introduction

Obesity is a global health issue characterized by an excessive body weight (i.e., body mass index (BMI) ≥ 30 kg/m^2^) and accompanying metabolic disorders, such as type 2 diabetes and cardiovascular disease [1]. Obesity is primarily caused by a chronic energy imbalance, often resulting from increased consumption of energy-dense foods that are rich in fat and sugars [1]. Accordingly, a better understanding of the mechanisms controlling food intake is essential for the development of strategies for the treatment of obesity. In this context, research focused on gastrointestinal hormones that modulate appetite and energy metabolism has exponentially increased.

Ghrelin is a gastrointestinal hormone with an important role in body energy homeostasis, stimulating food intake and adiposity [2,3]. Ghrelin is primarily produced by P/D1 (human) and X/A-like (rodent) cells of the stomach [4], and requires a number of enzymatic steps to become biologically active. Initially, cleavage of pre-proghrelin produces proghrelin, which subsequently undergoes another cleavage step at the Pro-Arg site on the C-terminal region, by the enzyme prohormone convertase 1/3 (PC1/3), to produce the mature ghrelin peptide [5,6]. In addition, acylation of ghrelin, on its third serine residue, by the enzyme ghrelin O-acyltransferase (GOAT), results in the production of acyl ghrelin [6,7,8], which can bind the ghrelin receptor, growth hormone secretagogue receptor 1a (GHSR1a) [6,9]. Acyl ghrelin accounts for less than 10% of the circulating levels of total ghrelin, while 90% is found as des-acyl ghrelin [10,11,12].

It is well established that circulating ghrelin levels fall after food intake [13,14,15]. Multiple peripheral mechanisms are involved in the postprandial reduction in circulating ghrelin levels, including digestive breakdown of nutrients [16] and the increase in circulating levels of ghrelin-inhibiting hormones (e.g., cholecystokinin [17] and insulin [18,19]). Furthermore, there is emerging evidence to indicate that gastric nutrient-sensing is an important contributing factor in the control of ghrelin secretion. Previous studies have demonstrated that numerous nutrient chemosensors are highly expressed in gastric ghrelin cells, including receptors for bitter (taste receptors type 2; T2Rs [20,21]), sweet and umami compounds (taste receptor 1 member 3; T1R3 [22,23]), short-chain fatty acids (free fatty acid receptor (FFAR) 2 [24]), long-chain fatty acids (FFAR4 [23,24] and cluster of differentiation (CD) 36 [23]), and protein digestion products (calcium-sensing receptor (CaSR [24,25]) and G-protein-coupled receptor 93 (GPR93 [23]). Many of these nutrient chemosensors were found to be involved in ghrelin secretion. Bitter compounds stimulated ghrelin secretion, via T2R5 and T2R10 activation, in gastric segments and cultures from humans [26]. In contrast, glucose reduced ghrelin release, via T1R3 activation, in gastric cultures from obese patients [26]. Similarly, FFAR2 agonists reduced ghrelin secretion from mouse gastric cultures [24]. However, the role of FFAR4 and CaSR in ghrelin release remains elusive, with reports showing that they can stimulate [24,27,28], reduce [24,29] or not affect [25,27] ghrelin secretion.

Disturbances in the ghrelin system, such as reduced circulating ghrelin levels [30,31,32], central ghrelin resistance [33], and inability of ghrelin to stimulate food intake [34], have been reported in obesity. However, there is limited knowledge available on the mechanisms that control ghrelin secretion in obesity. Regarding the role of nutrients as mediators of ghrelin secretion, previous reports have shown that obesity impairs ghrelin secretion in response to bitter and sweet stimuli in the small intestine [26] as well as gastric ghrelin secretion mediated by protein hydrolysates [25]. The aim of the current study was to determine, using a HFD-induced obese mouse model [35,36], obesity-induced changes in mRNA and protein expression of gastric nutrient chemosensors, with a particular emphasis on the degree of co-expression with gastric ghrelin cells.

## 2. Materials and Methods

### 2.1. Study Design and Ethics

Male C57BL/6 mice (7-weeks old) were group-housed with littermates for the duration of the experiment in a facility with light (12 h light/dark cycle), temperature (22 ± 0.5 °C) and humidity (40–60%) controlled environment. After a one-week acclimatization, mice were randomly assigned to 12 weeks of either a standard laboratory diet (SLD) or HFD, with ad libitum access to their respective diet and drinking water. The SLD contained 18%, 24% and 58% energy from fat, protein and carbohydrates (Teklad Rodent Diet 2018, ENVIGO, Madison, WI, USA), and the HFD contained 60%, 20% and 20% energy from fat (lard), protein and carbohydrates, respectively (adapted from Research Diets Inc., New Brunswick, NJ, USA). The body weight of animals was determined weekly. All experiments were approved by the South Australian Health & Medical Research Institute Animal Ethics Committee (SAM232, 1 December 2016).

### 2.2. Procedure for Tissue Collection

For gene expression experiments, mice were anesthetized by isoflurane inhalation (3% isoflurane in 1.5% oxygen) and exsanguinated for blood glucose measurement using a glucose meter (Accu-Chek, Castle Hill, NSW, Australia). The gonadal fat pad was excised and weighed. After removal of the stomach, mucosal scrapings of the corpus and antrum (i.e., glandular regions) were collected and snap-frozen in liquid nitrogen prior to storage (−80 °C) until required. For the immunohistochemistry experiments, mice were anesthetized by isoflurane inhalation. Once fully anesthetized, mice were given an i.p. injection of pentobarbitone (0.2 mL, 60 mg mL^−1^) immediately prior to transcardial perfusion of warm heparinised saline (flow rate: 17 mL min^−1^, 3 min) followed by cold 4% paraformaldehyde dissolved in 0.1 M phosphate buffer (PFA-PB, flow rate: 15 mL min^−1^, 50 mL). The stomach was removed, opened along the greater curvature and incubated in PFA-PB at room temperature for 2 h. After fixation, the stomach was placed in 30% sucrose-PB solution overnight for cryoprotection, prior to positioning and embedding in optimal cutting temperature compound (Tissue-Tek, ProSciTech, Kirwan, QLD, Australia) to enable longitudinal cryosectioning of the stomach. After processing, the tissue was stored at −80 °C until required for sectioning.

### 2.3. Measurement of Chemosensor mRNA and Protein Levels in the Mouse Stomach and Degree of Co-Expression with Ghrelin

#### 2.3.1. Quantitative RT-PCR

The detailed protocol for gene expression experiments has been previously reported [23]. In brief, a PureLink RNA Mini kit (Thermo Fisher Scientific, Adelaide, SA, Australia) was used to extract total RNA from the corpus and antrum mucosa, according to the manufacturer’s instructions. Spectrophotometry (260 nm) was used to quantify total RNA, and the A_260/280_ ratio was used as an estimation of purity.

An EXPRESS One-Step Superscript™ qRT-PCR Kit (Life Technologies, Adelaide, SA, Australia) and 7500 Fast Real-Time PCR system (Applied Biosystems, Adelaide, SA, Australia) was used for the quantitative real-time PCR (qRT-PCR) experiments. Predesigned TaqMan™-based assays (Thermo Fisher Scientific (Table 1)) for ghrelin, ghrelin-processing enzymes, chemosensors for fatty acids and proteins, as well as the sweet/umami receptor subunit T1R3 were used, with each assay run in triplicate. Three housekeeping genes, namely β-2 microglobulin (B2M), hypoxanthine-guanine phosphoribosyltransferase (HPRT) and peptidylprolyl isomerase A (PPIA), were used based on their averaged stability value (0.001) determined by NormFinder (Department of Molecular Medicine (MOMA), Aarhus University Hospital, Denmark). A further DNase digestion with ezDNase kit (Invitrogen, Adelaide SA, Australia) was performed to eliminate genomic DNA from total RNA samples. Negative controls were performed substituting RNA template with RNase-free water. Minus-reverse transcriptase controls were performed substituting reverse transcriptase with RNase-free water. Relative mRNA expression was calculated using the 2^−ΔCT^ method [37].

#### 2.3.2. Immunohistochemistry

The experimental conditions for the immunohistochemistry were adapted from a previous report [23]. Stomach cryosections (10 µm) were air-dried prior to three 5 min rinses in PBS (pH 7.4) containing 0.2% Triton X-100 (PBS-TX; Sigma-Aldrich, Castle Hill, NSW, Australia). The tissue was then blocked for 60 min at room temperature with PBS-TX containing 10% normal donkey serum (Sigma-Aldrich). This was followed by three washes with PBS-TX for 2 min. Goat anti-ghrelin (1:800, ab104307, Abcam, VIC, Australia) and rabbit anti-CaSR (1:100, NBP2-38622, Novus Biologicals, Centennial, CO, USA) antibodies were diluted in PBS-TX, and cryosections underwent overnight incubation at 4 °C. This was followed by a PBS-TX wash (3 times, 5 min) to remove unbound antibody. The cryosections were then incubated for 60 min at room temperature with donkey anti-rabbit conjugated to Alexa Fluor^®^ 488 or donkey anti-goat conjugated to Alexa Fluor^®^ 568 (Invitrogen) dissolved in PBS-TX (1:200). The cryosections were then rinsed with PBS-TX (3 times, 5 min) prior to mounting, using ProLong^®^ Diamond Antifade reagent with DAPI (Invitrogen), and coverslipping. Single label controls were run to confirm no bleed-through of fluorescence under different filters. No immunofluorescence was detected in slides where the primary antibody was omitted. The specificity of the ghrelin antibody was confirmed in double-labelling experiments in the mouse stomach, with goat anti-ghrelin immunoreactive cells displaying 95.2% co-localisation with a second rabbit anti-ghrelin antibody (1:1600, ab129383, Abcam). Specific immunolabelling of the CaSR antibody was previously tested in the parathyroid gland and kidney [38].

#### 2.3.3. Microscopy, Imaging and Cell Quantification

Immunolabelling was visualized using a BX51 epifluorescence microscope (Olympus, Parkside, SA, Australia) equipped with narrow filters for Alexa Fluor^®^ 488 and 568. An XM10 monochrome camera (Olympus, Parkside, SA, Australia) was used to acquire the images. CellSens Dimensions Imaging Software (Olympus, Parkside, SA, Australia) was used to adjust the brightness and contrast.

Cell counts were performed in 5–6 non-consecutive sections per gastric region and animal. Immunopositive cells were manually counted in a 159 × 159-µm square area at the base of the glandular region (i.e., location of gastric ghrelin [39] and CaSR [40] immunopositive cells) using the software FIJI [41].

### 2.4. Statistical Analysis

Data are expressed as mean ± SEM. All statistical analysis was performed using GraphPad Prism software version 7.02 (La Jolla California, USA). Differences in gonadal fat mass, blood glucose levels and the density of CaSR-immunopositive cells in gastric antrum between SLD and HFD groups were evaluated by unpaired Student’s *t*-test. Differences in weekly body weight, mRNA expression, density of ghrelin-immunopositive cells, and percentage of co-expression of ghrelin and CaSR were determined by two-way ANOVA followed by Sidak post hoc test. Statistical significance for unpaired Student’s *t*-test and two-way ANOVA is defined as * *p* < 0.05, ** *p* < 0.01 and *** *p* < 0.001. Statistical significance for Sidak post hoc test is indicated as ^#^ *p* < 0.05, ^§^ *p* < 0.01 and ^^^ *p* < 0.001.

## 3. Results

### 3.1. Metabolic Parameters in SLD and HFD Mice

**Figure 1:**
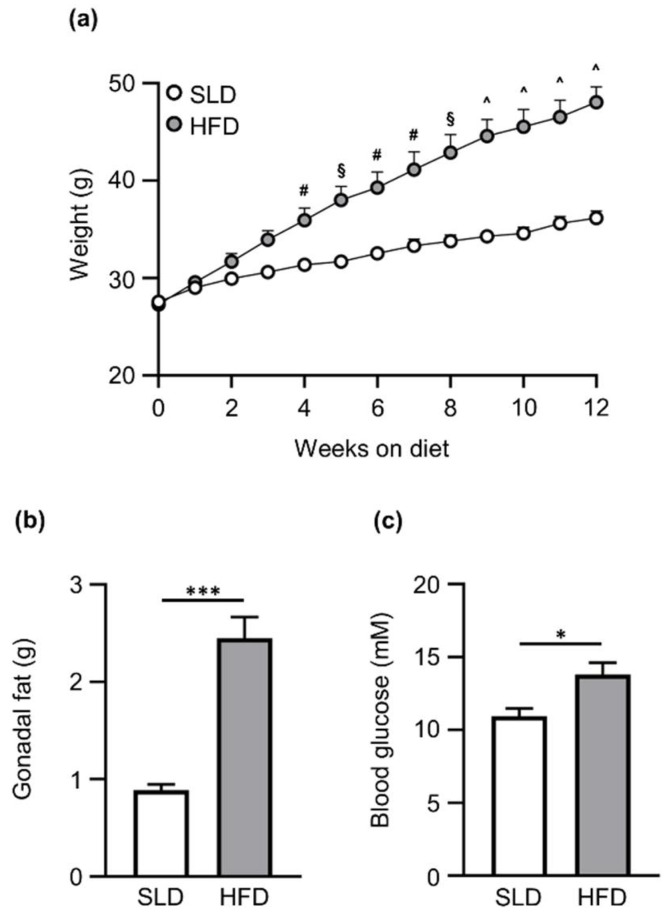
Metabolic parameters of C57BL/6 mice on a standard laboratory diet (SLD) or high-fat diet (HFD) for 12 weeks. (**a**) HFD mice (*n* = 14) gained significantly more weight than SLD-fed mice (*n* = 14). HFD mice presented higher (**b**) fat mass and (**c**) blood glucose levels than SLD mice after 12 weeks in the diet (*n* = 7/group). Data are expressed as mean ± SEM. Two-way ANOVA followed by Sidak post hoc test was used to determine the differences in body weight, and unpaired Student’s *t*-test was used to assess differences in gonadal fat mass and blood glucose levels. Statistical significance for two-way ANOVA and unpaired Student’s *t*-test is defined as * *p* < 0.05 and *** *p* < 0.001. Statistical significance for Sidak post hoc test is denoted as ^#^ *p* < 0.05, ^§^ *p* < 0.01 and ^ *p* < 0.001.

Weekly weight, gonadal fat mass and blood glucose levels are illustrated in Figure 1. HFD-fed mice weighed significantly more than SLD-fed mice by the end of the experiment (Figure 1a; HFD: 48.1 ± 1.6 g vs. SLD: 36.2 ± 0.7 g; diet effect, *p* ˂ 0.001; time effect, *p* ˂ 0.001; interaction, *p* ˂ 0.001). Gonadal fat pad mass was significantly increased in HFD mice compared to SLD mice after 12 weeks of diet (HFD: 2.5 ± 0.2 g vs. SLD: 0.9 ± 0.05 g; *p* ˂ 0.001; Figure 1b). Furthermore, ad libitum-fed blood glucose levels at week 12 were significantly higher in HFD mice compared to SLD mice (HFD: 13.8 ± 0.8 mM vs. SLD: 10.9 ± 0.5 mM; *p* ˂ 0.05; Figure 1c).

### 3.2. Gastric Nutrient Chemosensors, Ghrelin and Ghrelin-Processing Enzyme mRNA Expression in SLD and HFD Mice

**Figure 2:**
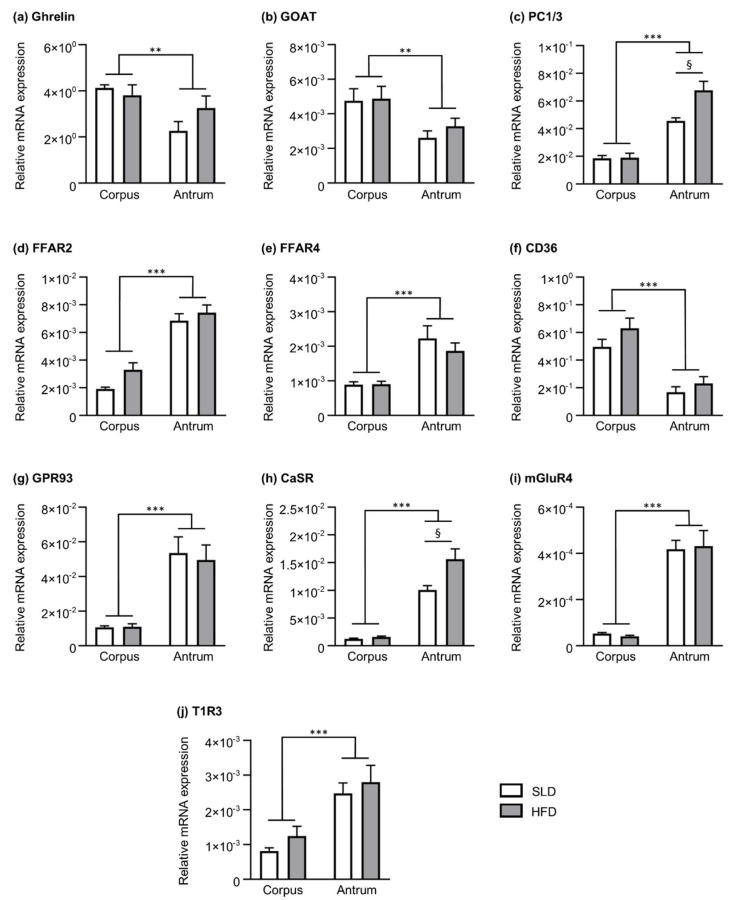
Nutrient chemosensors, ghrelin and ghrelin-processing enzymes relative mRNA expression in the corpus and antrum of a 12-week standard laboratory diet-fed (SLD, *n* = 5) or high-fat diet-fed (HFD, *n* = 5–7) mice. Relative mRNA expression of (**a**) ghrelin, the ghrelin-processing enzymes, (**b**) GOAT and (**c**) PC1/3, and the nutrient chemosensors for the detection of (**d**–**f**) fatty acids, (**g**–**i**) protein digestion products and (**j**) sweet and umami taste. The expression is relative to the average housekeeper values for PPIA, HPRT and B2M. Data are expressed as mean ± SEM. Differences in gene expression were determined by two-way ANOVA followed by Sidak post hoc test. Statistical significance for two-way ANOVA is defined as ** *p* < 0.01 and *** *p* < 0.001. Statistical significance for Sidak post hoc test is denoted as ^§^ *p* < 0.01.

Relative mRNA levels of nutrient chemosensors, ghrelin and enzymes involved in ghrelin production in the corpus and antrum of SLD and HFD mice are shown in Figure 2. Transcript levels of ghrelin (Figure 2a) and GOAT (Figure 2b) were higher in corpus compared to antrum (region effect, *p* ˂ 0.01); however, they remained unchanged by HFD. In contrast, mRNA expression of PC1/3 (Figure 2c) was higher in the antrum compared to the corpus (region effect, *p* ˂ 0.001). Further, there was a diet effect (*p* ˂ 0.05) and an interaction (*p* ˂ 0.05) due to an increase of PC1/3 expression in the gastric antrum of HFD mice (1.5-fold higher, *p* ˂ 0.01).

The mRNA expression of fatty acid receptors FFAR2 (Figure 2d) and FFAR4 (Figure 2e) was higher in the antrum than corpus (region effect for both receptors, *p* ˂ 0.001), however there was no diet effect. In contrast, the expression of the fatty acid transporter, CD36 (Figure 2f), was lower in the antrum compared to the corpus (region effect, *p* ˂ 0.001), with no difference between SLD and HFD mice.

The mRNA expression of receptors for protein digestion products was higher in the antrum compared to the corpus for GPR93 (region effect, *p* ˂ 0.001; Figure 2g), CaSR (region effect, *p* ˂ 0.001; Figure 2h) and mGluR4 (region effect, *p* ˂ 0.001; Figure 2i). Although there was no diet effect on mRNA transcript levels of GPR93 and mGluR4, there was a diet effect (*p* ˂ 0.01) and interaction (*p* ˂ 0.05) due to the increase in antral CaSR in HFD mice (1.6-fold higher; *p* ˂ 0.01).

The mRNA levels of T1R3 (sweet and umami receptor subunit) were higher in the antrum compared to the corpus (Figure 2j; region effect, *p* ˂ 0.001), with no significant effect of HFD in the expression of this chemosensor.

### 3.3. The Density of Ghrelin and CaSR Immunopositive Cells in SLD and HFD Mice

**Figure 3:**
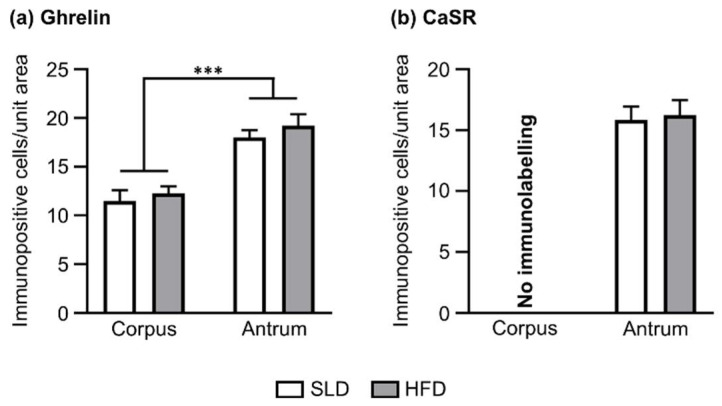
Density of ghrelin and CaSR immunopositive cells in the glandular base of gastric corpus and antrum of mice fed standard laboratory diet (SLD, *n* = 5) or high-fat diet (HFD, *n* = 5) for 12 weeks. (**a**) Cell counts showed a higher density of ghrelin cells in the glandular base of the gastric antrum than corpus. A comparable number of ghrelin cells was observed in the corpus of SLD and HFD mice. Similarly, comparable numbers of ghrelin cells were found in the antrum of SLD and HFD mice. (**b**) CaSR cells were located in the antrum only, with no immunolabelling observed in the corpus. A comparable number of CaSR cells were observed in the antrum of SLD and HFD mice. Cell counts are the mean value of 5–6 non-adjacent tissue sections per mouse and gastric region (unit area = 159 × 159 µm). Two-way ANOVA followed by Sidak post hoc test was used to assess the differences in the number of ghrelin cells, and unpaired Student’s *t*-test was used to assess differences in the number of CaSR cells. *** *p* < 0.001.

Immunofluorescence studies were performed to assess the HFD-induced changes in the density of ghrelin immunopositive cells. Further, due to the obesity-induced increase in antral CaSR mRNA expression, the density of CaSR immunopositive cells was also determined. The number of ghrelin-positive cells was higher in the antrum (SLD antrum: 18.0 ± 0.8 cells/unit area; HFD antrum: 19.2 ± 1.2 cells/unit area) compared to the corpus (SLD corpus: 11.5 ± 1.1 cells/unit area; HFD corpus: 12.3 ± 0.7 cells/unit area; *p* ˂ 0.001; Figure 3a); a finding that was associated with the higher density of ghrelin cells/unit area in the glandular base of the antrum compared to the corpus (Supplementary Figure S1). Furthermore, the density of ghrelin-positive cells in both gastric regions remained stable in SLD and HFD conditions. Immunolabelling for CaSR was observed in the gastric antrum only, with no change in the density of CaSR-positive cells in HFD compared to SLD mice (SLD antrum: 15.8 ± 1.1 cells/unit area vs. HFD antrum: 16.2 ± 1.2 cells/unit area; Figure 3b).

### 3.4. Co-Expression of Ghrelin and CaSR in the Gastric Antrum of SLD and HFD Mice

**Figure 4:**
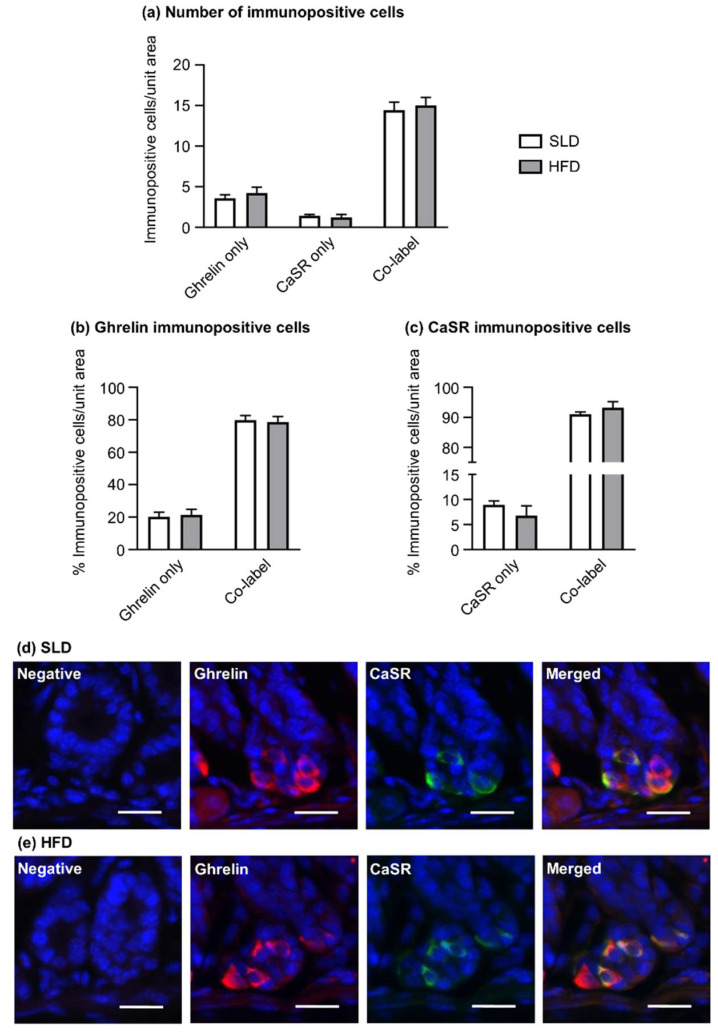
Co-expression of ghrelin and CaSR in the antrum of mice fed standard laboratory diet (SLD, *n* = 5) and high-fat diet (HFD, *n* = 5) for 12 weeks. (**a**) Most immunopositive cells co-expressed ghrelin and CaSR. (**b**) Approximately 80% of ghrelin immunopositive cells expressed CaSR in SLD and HFD-fed mice, (**c**) and over 90% of CaSR immunopositive cells contained ghrelin, independently of the diet. (**d**,**e**) Images of ghrelin and CaSR co-expression in the gastric antrum of SLD and HFD mice. Cell counts are the mean counts of 5–6 tissue sections per mouse and gastric region (unit area = 159 × 159 µm). Data are expressed as mean ± SEM. Statistical significance was determined by two-way ANOVA followed by Sidak post hoc test.

Dual immunofluorescence experiments of ghrelin and CaSR were performed to determine the expression of CaSR within ghrelin cells of the gastric antrum of SLD and HFD mice. The majority of gastric antral immunopositive cells were found to co-express ghrelin and CaSR in SLD (14.4 ± 1.0 cells/unit area) and HFD mice (15.0 ± 1.0 cells/unit area; Figure 4a,d,e). Accordingly, nearly 80% of ghrelin immunopositive cells in the glandular base of antrum expressed CaSR, with no difference between SLD (79.8 ± 2.8% co-localization) and HFD mice (78.7 ± 3.4% co-localization; Figure 4b). Over 90% of CaSR immunopositive cells contained ghrelin, irrespectively of the type of diet (SLD: 91.2 ± 0.8% co-localization vs. HFD: 93.2 ± 2.0% co-localization; Figure 4c).

## 4. Discussion

The current study found that HFD-induced obesity did not alter the mRNA expression of ghrelin and a wide range of nutrient chemosensors in the mouse stomach. However, antral CaSR mRNA levels were augmented after chronic HFD feeding. Mechanistic studies have shown the involvement of CaSR in the modulation of acyl ghrelin secretion [24,28]. Therefore, this report further investigated the changes in the number of CaSR and ghrelin immunopositive cells, as well as their co-expression in HFD-induced obesity, and showed that HFD-induced obesity did not affect the density of CaSR and ghrelin immunopositive cells in the mouse stomach. Furthermore, CaSR was highly expressed within the antral ghrelin cell population of lean and HFD-induced obese mice in comparable proportions.

The expression of nutrient receptors in the mouse stomach is known to be regional, with a previous report showing that a large repertoire of gastric nutrient chemosensors are highly expressed in the antrum compared to the corpus [23]. The current study confirmed these findings, showing that most chemosensors presented higher mRNA expression in the antrum compared to the corpus, with the exception of CD36. The higher expression of CD36 in the gastric corpus may be explained by the enrichment of this fatty acid transporter in corpus-predominant parietal [23,42] and ghrelin cells [23]. Furthermore, this report extends previous knowledge on the expression of gastric nutrient chemosensors, by defining changes in their mRNA levels in a well-established diet-induced obese mouse model [35,36] with elevated body weight, fat mass and blood glucose levels. HFD mice presented an increased mRNA expression of PC1/3 and CaSR compared to SLD mice, specifically in the gastric antrum. PC1/3 is responsible for catalyzing the conversion of proghrelin into mature ghrelin [5,43]; however, this enzyme is also involved in the proteolytic cleavage of multiple prohormones, including prosomatostatin [43,44] and progastrin [43,45]. Ghrelin levels are known to be decreased in obesity [46,47,48], while the density of somatostatin-producing cells remains unchanged in the gastric mucosa of obese mice [47], and plasma gastrin levels are increased in HFD mice [49]. Therefore, it is possible that the increase of PC1/3 mRNA expression in the gastric antrum is responsible for the increased plasma gastrin levels [49], rather than the reduced ghrelin levels observed in HFD mice [47]. However, studies are required to elucidate the effect of increased PC1/3 on gastrin and ghrelin secretion in HFD-induced obesity.

It is well established that circulating ghrelin levels are reduced in obesity [46,47,48]. The current study shows no significant difference in ghrelin mRNA levels and density of ghrelin immunopositive cells in HFD-induced obese mice compared to control mice. Previous reports have shown similar findings in lean and obese humans [26,50], as well as SLD and HFD rats [51]. Additionally, it has been demonstrated in human and rodent studies that the obesity-dependent reduction of ghrelin secretion occurs despite unchanged [26], decreased [33] and increased [47] ghrelin mRNA levels and density of ghrelin cells. This suggests that these fluctuating parameters may not be a determining factor for the reduction of ghrelin secretion in obesity. Moreover, most reports have shown a stable expression of GOAT in obesity [35,52,53]. Consistent with these findings, the current study shows that GOAT mRNA levels remained unchanged in HFD-induced obese mice compared to SLD mice.

The mRNA expression of the CaSR is known to peak in the antrum of humans [25] and mice [23,40], in comparison to other gastric regions. In agreement with previous studies [23,40], this report demonstrated higher CaSR mRNA levels in the antrum compared to the corpus, and an antral-specific immunolabelling. There is minimal information available on the expression of gastric CaSR in obesity, with the current report showing an increase in antral CaSR mRNA expression in HFD-induced obese mice compared to controls. In contrast, a previous report, using gastric tissue from obese humans, showed a region-specific downregulation of antral CaSR mRNA levels [25]. It is known that gene expression of nutrient chemosensors is modulated by fasting and feeding [54,55], diet [56,57,58] and grade of obesity [59]. While the current study showed CaSR mRNA expression from ad libitum-fed mice, the human study presented CaSR mRNA levels from fasted obese individuals and organ donors [25]. Consequently, the contrasting results may reflect differences in feeding state or simply species variations. Moreover, these findings demonstrate a region-specific alteration in the human and murine antral CaSR mRNA expression under chronic excess energy intake, and call for further research to determine if changes in CaSR in HFD-induced obesity are associated with antral impairment of gastric hormone secretion, such as ghrelin.

CaSR is a chemosensor for calcium [60], aromatic amino acids [60] and protein hydrolysates [61], which when activated stimulates the secretion of multiple gut hormones, including gastrin [62], cholecystokinin [63] and glucagon-like peptide 1 [61]. However, the role of CaSR in ghrelin secretion is less established, with reports showing that activation of CaSR results in an increase [24,28] or decrease [24] in acyl ghrelin secretion. For example, mechanistic experiments using mouse gastric mucosal cells have shown that only supraphysiological concentrations of the CaSR agonist, CaCl_2_ (40 mM), significantly decrease acyl ghrelin release [24]. Similarly, the CaSR positive allosteric modulator, R-568, decreased acyl ghrelin secretion in the presence of 1.8 mM CaCl_2_ in the cell culture media. However, this effect was shifted to stimulation when R-568 was tested under higher CaCl_2_ concentrations that were initially ineffective in the mobilisation of acyl ghrelin secretion (4 mM) [24]. These results illustrate the complex nature of CaSR, which has different ligand binding sites [64,65] that, upon activation, are able to couple numerous G proteins and downstream pathways that interact to contribute to cooperative responses [65,66], plausibly modulating the decrease or increase of acyl ghrelin secretion. Previous reports have demonstrated that peptones stimulate acyl ghrelin secretion from gastric mucosal segments from humans [25] and mice [28]. In contrast, peptones down-regulate total ghrelin secretion (i.e., containing mostly des-acyl ghrelin) from human gastric mucosal segments [25]. Mechanistic studies using the ghrelinoma MGN3-1 cell line have shown that the peptone-induced secretion of acyl ghrelin is partially reversed by the CaSR negative allosteric modulator, calhex-23 [28]. However, the peptone-induced inhibition of total ghrelin secretion from human mucosal segments is unchanged by the negative allosteric modulator, NPS-2143 [25]. Overall, these findings suggest that acyl and des-acyl ghrelin secretion are independently modulated, with CaSR possibly only participating in the control of acyl ghrelin secretion. In agreement with this, the current report established a high degree of ghrelin and CaSR co-expression in the antrum of the mouse stomach, which suggests a direct role of CaSR in the control of ghrelin secretion from antral ghrelin cells. Furthermore, the number of CaSR immunopositive cells and co-expression with ghrelin were comparable in lean and HFD-induced obese mice. Therefore, the characteristic reduction in ghrelin secretion in obesity [46,47,48] is not due to changes in the number of CaSR immunopositive cells and/or the degree of co-expression of ghrelin and CaSR. However, as a limitation of the current study, quantitative protein expression of CaSR was not measured. Therefore, whether the protein level of CaSR is altered in HFD-induced obesity and responsible for the reduction in ghrelin secretion in obesity requires further investigation. Moreover, species differences in the expression of nutrient chemosensors are possible. Accordingly, a careful comparison between rodent and human studies is necessary.

## 5. Conclusions

This study provided information on the expression of nutrient chemosensors and ghrelin in a diet-induced obese mouse model. Most nutrient chemosensors investigated in this report had a comparable mRNA expression in lean and HFD-induced obese mice. Similarly, ghrelin mRNA expression and density of ghrelin immunopositive cells remained unchanged in HFD mice, indicating that reduced circulating levels of ghrelin in obesity are caused by a reduction in ghrelin protein production and/or secretion. Gastric nutrient chemosensors are important contributors to the modulation of ghrelin secretion, which is critical for the regulation of food intake and energy homeostasis. Available research is contradictory and indicates that CaSR is a signaling mechanism for the reduction [24] and stimulation [24,28] of acyl ghrelin secretion. This report established a high co-expression of CaSR and ghrelin in the gastric antrum, suggesting a direct role of this chemosensor in the antral secretion of acyl ghrelin. Circulating ghrelin levels are reduced in obesity and, therefore, it is possible that the increase in antral CaSR mRNA in HFD-induced obesity may impact acyl ghrelin secretion. However, further research is required to confirm translation at the protein level and how this may impact on gastric ghrelin secretion in obesity.

## Figures and Tables

**Table 1:** Description and product code of qRT-PCR primers.

Target	Description	TaqMan™ Assay ID
Ghrelin	Gastrointestinal hormone	Mm00445450_m1
GOAT	Ghrelin-processing enzyme	Mm01200389_m1
PC1/3	Ghrelin-processing enzyme	Mm00479023_m1
FFAR2 (GPR43)	Short-chain fatty acid receptor	Mm02620654_s1
FFAR4 (GPR120)	Long-chain fatty acid receptor	Mm00725193_m1
CD36 (FAT)	Fatty acid translocase	Mm00432403_m1
GPR93 (GPR92)	Protein hydrolysate receptor	Mm02621109_s1
CaSR	Calcium and aromatic amino acids receptor	Mm00443375_m1
mGluR4	Glutamate receptor	Mm01306128_m1
T1R3	Sweet and umami taste receptor subunit	Mm00473459_g1
B2M	Housekeeping gene	Mm00437762_m1
HPRT	Housekeeping gene	Mm01545399_m1
PPIA	Housekeeping gene	Mm02342429_g1

## Supplementary Materials

The following are available online at https://www.mdpi.com/2072-6643/12/9/2493/s1, Figure S1: The gastric corpus presents a higher number of ghrelin immunopositive cells compared to the antrum.
