# Supplementary material for: The Effect of High-Fat Diet-Induced Obesity on the Expression of Nutrient Chemosensors in the Mouse Stomach and the Gastric Ghrelin Cell
Source: Nutrients. 2020 Aug 19;12(9):2493. doi: 10.3390/nu12092493 (PMC7551456; doi:10.3390/nu12092493)
Supplement: Supplementary file 1 [file nutrients-12-02493-s001.pdf]

Supplementary Materials: The following are available online at www.mdpi.com/xxx/s1, Figure S1: The gastric corpus presents a higher number of ghrelin immunopositive cells compared to the antrum.

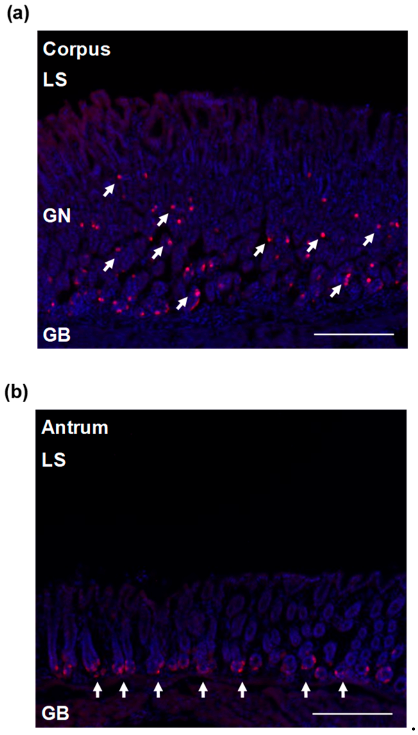

Supplementary figure 1. The gastric corpus presents a higher number of ghrelin immunopositive cells compared to the antrum. (a) While the corpus presents numerous ghrelin cells uniformly distributed throughout the glandular base and neck of the tissue, (b) the gastric antrum presents a high density of ghrelin cells located in the glandular base of the tissue only. Abbreviations: LS, luminal surface; GN, glandular neck; GB, glandular base. Scale bars = 200μm.
